# The Role of Digital Tools and Their Implementation Within Patient Care Pathways for Rare Brain Disorders: The Case of Phenylketonuria

**DOI:** 10.1111/ene.70575

**Published:** 2026-04-09

**Authors:** Sara Cannizzo, Vinciane Quoidbach, Bernadette Sheehan‐Gilroy, Tobias Hagedorn, Agata Bak, Suzanne L. Dickson, Eileen P. Treacy, Alvaro Hermida, Anita MacDonald, Eva Venegas, James O'Byrne, Maurizio Scarpa, Francjan Van Spronsen, Eric Lange, Tim Buckinx, Ahmad Monavari, Stephan vom Dahl, Leopoldo Trieste, Giuseppe Turchetti

**Affiliations:** ^1^ Scuola Superiore Sant'anna Istituto di Management Pisa Italy; ^2^ European Brain Council Brussels Belgium; ^3^ Munster Technological University Kerry Campus Tralee Ireland; ^4^ German PKU and Allied Disorders Patients Association DIG PKU Fürth Germany; ^5^ European Society for Phenylketonuria & Allied Disorders Hiddenhausen Germany; ^6^ Universidad Nacional de Educación a Distancia Madird Spain; ^7^ Federación Española de Enfermedades Metabólicas Hereditarias Madrid Spain; ^8^ Institute of Neuroscience and Physiology The University of Gothenburg Gothenburg Sweden; ^9^ School of Medicine Trinity College Dublin Ireland; ^10^ Universidad de Santiago de Compostela Santiago de Compostela Spain; ^11^ Dietetic Department Birmingham Children's Hospital Birmingham UK; ^12^ Endocrinology Department Hospital Virgen del Rocío Sevilla Spain; ^13^ MetabERN, European Reference Network for Hereditary Metabolic Diseases Udine Italy; ^14^ National Centre for Inherited Metabolic Disorders Mater Misericordiae University Hospital Dublin Ireland; ^15^ Centre for Rare Diseases Udine University Hospital Udine Italy; ^16^ Beatrix Children's Hospital University Medical Centre of Groningen Groningen the Netherlands; ^17^ Epihunter Farow Hasselt Belgium; ^18^ Klinik f. Gastroenterologie, Hepatologie und Infektiologie Universitätsklinikum Düsseldorf Düsseldorf Germany

**Keywords:** digital patient care pathways, European Reference Networks for rare diseases, healthcare professionals' expert opinion, patients' perspectives, phenylketonuria

## Abstract

**Introduction:**

The COVID‐19 pandemic accelerated the adoption of digital health solutions in healthcare. Phenylketonuria (PKU) is a rare condition requiring chronic management and frequent assessments, making it a useful model for examining how digital health tools support patient and caregiver education, communication with healthcare professionals and facilities, and patient care pathways.

**Methods:**

Patient representatives and expert clinicians developed qualitative, co‐designed ad hoc surveys during virtual workshops. From October 2023 to March 2024, the surveys were available online through EUSurvey English, Spanish, and German, and distributed to PKU patients in Spain, Germany, and Ireland by national PKU patient associations.

**Results:**

The survey co‐design process identified crucial topics significant to key stakeholders in rare disease management. Diverse perspectives emerged on the roles and utility of digital tools: (1) rare disease patients may prefer hybrid care models combining face‐to‐face and digital interactions; (2) digital tools were perceived as particularly useful for supporting information exchange, education, preparation for clinical visits, and patient engagement.

**Conclusions:**

This paper examines unmet needs in digital care pathways for PKU from the perspectives of patients, caregivers, and clinicians. Findings provide important insights into the needs of patients with rare diseases and the most effective channels for engaging and communicating with them. Although clinical and cost‐effectiveness were not evaluated, these findings could guide future research and policy discussions on incorporating digital solutions into rare diseases patient care pathways.

## Graphical abstract

This infographic summarizes the study on the perceived role of digital tools in supporting information, education, and communication in phenylketonuria (PKU) care. Survey findings from patients, caregivers, and healthcare professionals highlight a preference for hybrid care pathways that integrate digital solutions with face‐to‐face consultations across different phases of the PKU patient care pathway.

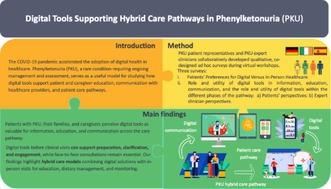

## 1 Introduction

Phenylketonuria (PKU) [1] is an autosomal recessive disorder caused by a deficiency of phenylalanine hydroxylase (PAH) [2, 3]. If untreated, PKU can lead to impaired cognitive development due to hyperphenylalaninemia. Early diagnosis is vital, as PKU is manageable with a low phenylalanine diet, Phe‐free L‐amino acid supplements, or low Phe glycomacropeptide [4].

PKU has a birth frequency in Europe of approximately 1 in 10,000, which varies geographically [5]. In Ireland, the prevalence is estimated at 1 in 4500, in Germany at 1 in 5700, in Spain at 1 in 10,115, and the lowest in Northern Europe is in Finland at 1 in 112,000 [6]. PKU is reliably detected through newborn screening programs. Children with positive results are referred to specialized metabolic diagnostic procedures and treatment to ensure optimal outcomes. Treatment should start before 10 days of age. Current European guidelines recommend lifelong treatment to keep blood Phe levels within the target range based on patient age [3, 7, 8]. The primary treatment goal is to maintain normal neurocognitive and psychosocial functioning. Monitoring and lifelong follow‐up of individuals with PKU include frequent laboratory analyses of Phe blood levels. Outpatient visits in multidisciplinary metabolic units may involve micronutrient assessments, nutritional counseling, neurocognitive testing, bone density measurements, early detection of comorbidities, evaluations of executive and psychosocial functioning, well‐being, quality of life, and assessments of adaptive issues such as behavioral problems, maternal PKU, and other age‐specific investigations.

Complementary guidelines suggest educating PKU patients about low protein intake is vital for lifelong self‐care and optimal metabolic control [9]. Education on dietary treatment, self‐care, and consistent follow‐up is crucial at any age, particularly during adolescence when blood Phe control risks worsening.

The COVID‐19 pandemic accelerated healthcare's digital transformation. Telemedicine services expanded, shifting many healthcare services online [10, 11]. Telehealth has proven valuable for improving treatment adherence and offering better, continuous care for patients with PKU [12]. As defined by the World Health Organization “digital health” refers to the use of technology to enhance people's health and well‐being at individual and population levels and to improve patient care through intelligent processing of clinical and genetic data [13, 14, 15].

The present study aimed to investigate the perspectives of patients and caregivers regarding whether digital health tools might help inform and educate patients and their caregivers, and enhance communication among patients, their caregivers, and healthcare professionals or healthcare centers. Our starting point is our previous Value of Treatment for Brain Disorders (VOT) project, which aimed to define a reference care pathway for organizing healthcare services and involved PKU patient representatives and European PKU expert clinicians [16, 17]. Additionally, the current study aims to determine during which phases of the care pathway, as viewed by patients, caregivers, and healthcare professionals, a structured inclusion and implementation of digital health tools could be considered to improve information, education, and communication. We present and discuss PKU as a valuable example of a rare condition requiring chronic management with frequent assessments.

## 2 Methods

The Digital Care Pathways for Rare Brain Diseases research project (2023–2024) was coordinated by the European Brain Council (EBC) in collaboration with academic partners and PKU Patients' Associations from Germany, Ireland, and Spain [18, 19].

Key stakeholders were engaged from the very beginning of the project in the co‐design of the surveys. Three virtual stakeholders' workshops were organized; the first was held in May 2023, involving patients with PKU and their representatives, European PKU expert clinicians from renowned clinical centers, members of the European Reference Networks for rare diseases (ERNs), specifically MetabERN [20, 21]. A second online workshop took place in June 2023, targeting patients with PKU and their representatives, to introduce the co‐design process for defining the structure and content of the survey. The third online workshop was held in May 2024, after the data collection, to present and discuss the preliminary results of the patients' PKU perspective survey and the healthcare professionals' questionnaire.

The main goal of the survey was to understand if and how digital tools can support information, communication, and education in managing PKU throughout the different phases of the patient care pathway. The co‐design working group included three expert patient representatives from national PKU patient associations from Germany, Spain, and Ireland, two academics from Scuola Superiore Sant'Anna (Pisa, Italy), and the project manager coordinating the Digital Care Pathways for Rare Brain Diseases research project of EBC. PKU patients' representatives were involved in three co‐design online workshops conducted, one in July 2023 and two in September 2023, respectively. Patients reviewed questions to ensure clear, shared language, creating a comprehensive list that targeted patients with PKU, their families, and caregivers and were involved in the translation of the English version into Spanish and German. The survey was conducted online via the EU Survey [22] between October 15, 2023, and December 16, 2023.

Participation in the survey was voluntary, and data were collected anonymously. Moreover, the respondents completed a clear consent statement; thus, the institutional review board (IRB) was not requested.

European PKU expert clinicians and working group members adapted the survey for different targets during an online meeting in February 2024. The final version was available online in English via the EU Survey from February 20 to March 18, 2024, promoted by healthcare professionals from Spain, Germany, and Ireland with expertise in metabolic disorders. Using the same structure, the two surveys—one targeting PKU patients and caregivers and the other targeting healthcare professionals involved in PKU care management—comprise 43 questions divided into four sections: Section 1. Socio‐demographic data (eight single‐choice questions); Section 2. Information, Communication, and Educational Needs (six single‐choice, six multiple‐choice, and four open‐ended questions, allowing respondents to share additional perspectives); Section 3. Access, Diagnosis, Treatment, and Monitoring of PKU (nine single‐choice, seven multiple‐choice, and two open‐ended questions); and Section 4 consisting of one open‐ended question.

A third survey was conducted among PKU patients from Spain, Germany, and Ireland to share their preferences for digital versus in‐person healthcare services.

The main results of these three surveys are presented in the following section.

## 3 Results

### 3.1 Patients' Preferences Regarding Digital Versus In‐Person Healthcare

One hundred seventy‐one members of the PKU community (Germany: 117, Ireland: 33, Spain: 21) responded to a complementary tri‐national survey coordinated by three patient representatives. This survey explored the perspectives of patients, families, and caregivers on digital health tools, focusing on the importance, utility, and preference between digital and in‐person healthcare. Importance and usefulness were assessed on a 10‐point scale (1 = lowest; 10 = highest), showing slightly higher mean scores for in‐person care (importance 8.96; usefulness 8.69) compared with digital healthcare (mean 7.91 for both); however, when both options were available, 38% of respondents preferred an equal combination of in‐person and digital healthcare, supporting a hybrid model. Only a small minority would prioritize one of the two healthcare models over the other. For this minority, there would be a strong correlation between their preference and the distance to the nearest metabolic center. There was no correlation between the distance to the metabolic clinic and the prioritization of digital versus in‐person healthcare. Therefore, patients with PKU and their families are willing to travel for what they believe is the best possible care, regardless of the distance to their specialized metabolic center.

### 3.2 The Benefits of Digital Tools in Improving Information, Education, Communication, and the Role and Utility of Digital Tools Within the Different Phases of the PCP

#### 3.2.1 The Patients' Perspectives

A total of 75 respondents participated in the survey: Germany (*n* = 40, 53.3%), Ireland (*n* = 17, 22.7%), Spain (*n* = 15, 20%), and other countries (*n* = 3, 4%). Of them, 56% were female (*n* = 42), 48% (*n* = 36) were aged between 36 and 45 years, and 25.3% (*n* = 19) were aged between 21 and 35 years. Among the respondents, the number of patients with PKU was *n* = 44 (58.7%), and the number of caregivers/parents was *n* = 31 (41.3%).

Table 1 below offers a detailed overview of the patients responding to the survey by country: Germany (Table 1a), Ireland (Table 1b), and Spain (Table 1c).

**TABLE 1:** Overview of patients responding to the survey by country: Germany, Republic of Ireland, and Spain.

(A) Germany		
**PKU patients (adult)**	**Total *n* = 26**	
**Category**	***N***	**Distribution % (of *n* = 26)**
Age		
18–20	2	7.7%
21–35	7	26.9%
36–45	9	34.6%
46–60	8	30.8%
More than 60	0	0%
Gender		
Male	13	50%
Female	13	50%
Age at the diagnosis		
Newborn	23	88.5%
Child (3 months–7 years old)	3	11.5%
Treatment required		
Yes	26	100%
No	0	0%
On treatment at the time of the survey		
Yes	26	100%
No	0	0%
Followed by a metabolic specialized center		
Yes	24	92.3%
No	2	7.7%
**PKU patients (children)**	**Total *n* = 14**	
**Category**	***N***	**Distribution % (of *n* = 14)**
Age		
1–7	5	35.7%
8–11	4	28.6%
12–18	5	35.7%
Gender		
Male	7	50%
Female	7	50%
Age at the diagnosis		
Newborn	14	100%
Child (3 months–7 years old)	0	0%
Treatment required		
Yes	14	100%
No	0	0%
On treatment at the time of the survey		
Yes	14	100%
No	0	0%
Followed by a metabolic specialized center		
Yes	14	100%
No	0	0%

(B) Republic of Ireland		
**PKU patients (adult)**	**Total *n* = 9**	
**Category**	***N***	**Distribution % (of *n* = 9)**
Age		
18–20	1	11.1%
21–35	3	33.3%
36–45	4	44.4%
46–60	1	11.1%
More than 60		0%
Gender		
Male	6	66.7%
Female	3	33.3%
Age at the diagnosis		
Newborn	9	100%
Child (3 months–7 years old)	0	0%
Treatment required		
Yes	9	100%
No	0	0%
On treatment at the time of the survey		
Yes	8	88.9%
No	1	11.1%
Followed by a metabolic specialized center		
Yes	7	77.8%
No	2	22.2%
**PKU patients (children)**	**Total *n* = 8**	
**Category**	***N***	**Distribution % (of *n* = 8)**
Age		
1–7	2	25%
8–11	1	12.5%
12–18	5	62.5%
Gender		
Male	3	37.5%
Female	5	62.5%
Age at the diagnosis		
Newborn	8	100%
Child (3 months–7 years old)	0	0%
Treatment required		
Yes	8	100%
No	0	0%
On treatment at the time of the survey		
Yes	8	100%
No	0	0%
Followed by a metabolic specialized center		
Yes	8	100%
No	0	0%

(C) Spain		
**PKU patients (adult)**	**Total *n* = 8**	
**Category**	***N***	**Distribution % (of *n* = 8)**
Age		
18–20	1	12.5%
21–35	4	50%
36–45	3	37.5%
46–60	0	0%
More than 60	0	0%
Gender		
Male	5	62.5%
Female	3	37.5%
Age at the diagnosis		
Newborn	7	87.5%
Child (3 months–7 years old)	1	12.5%
Treatment required		
Yes	7	87.5%
No	1	12.5%
On treatment at the time of the survey		
Yes	8	100%
No	0	0%
Followed by a metabolic specialized center		
Yes	8	100%
No	0	0%
**PKU patients (children)**	**Total *n* = 7**	
**Category**	***N***	**Distribution % (of *n* = 7)**
Age		
1–7	5	71.4%
8–11	1	14.3%
12–18	1	14.3%
Gender		
Male	4	57.1%
Female	3	42.9%
Age at the diagnosis		
Newborn	7	100%
Child (3 months–7 years old)	0	0%
Treatment required		
Yes	6	85.7%
No	1	14.3%
On treatment at the time of the survey		
Yes	7	100%
No	0	0%
Followed by a metabolic specialized center		
Yes	7	100%
No	0	0%

Receiving reliable information about PKU and appropriate behaviors for disease self‐management is perceived as extremely important by most patients with PKU and their parents responding to the survey (58.7%). This information is specifically aimed at patients and their families (parents, partners, siblings, grandparents) and caregivers. Details about medical foods, new therapies, and accurate information about the disorder are valuable. Additionally, patients express interest in new clinical trial opportunities and the availability of a specialized metabolic center near their residence.

Parents of patients are eager to learn more about the potential impact of PKU on mental health, the long‐term risks associated with the disease, newborn screening programs, and the psychological implications of dietary restrictions, particularly for infants and toddlers. They are also interested in new therapies and research projects, updated texts and manuals on PKU self‐management, and patient support groups.

Patients indicated that communication among patients, families, caregivers, and healthcare professionals is extremely important (60%) or very important (37.3%) for managing PKU. This communication should be directed at patients and their families (parents, partners, siblings, grandparents), as well as other caregivers. Personalization of communication seems to be increasingly necessary, with preferences for individual sessions “in person,” “by telephone,” and “individual video calls” consistently viewed as helpful for routine communication. Additionally, virtual/video calls were recognized as a valuable option for remote interactions, aiding in maintaining continuity of care within a hybrid model.

**FIGURE 1:**
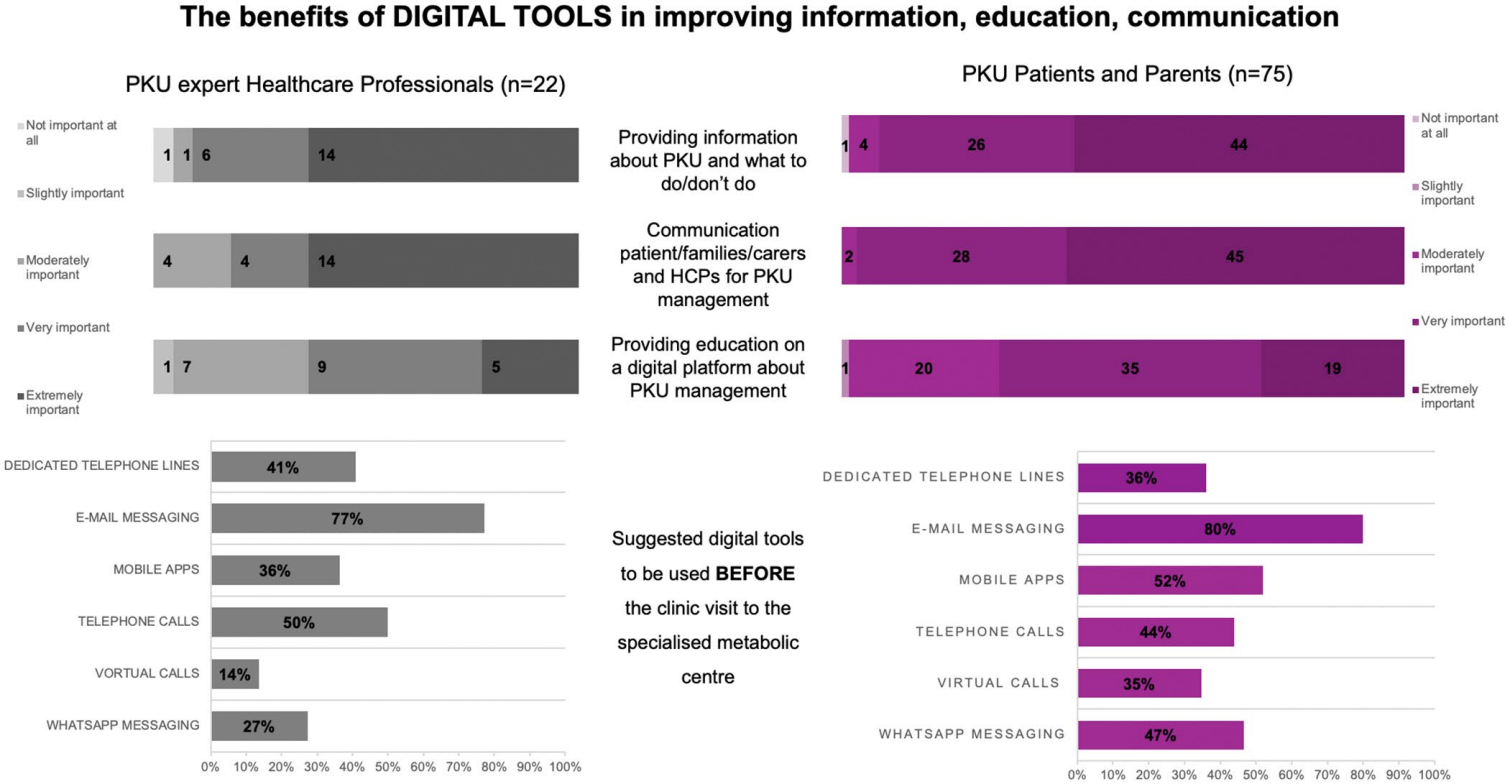
Benefits of digital tools in improving information, communication, and education. Differences and similarities between the HCP expert opinions and patients' and parents' points of view. *Source:* own elaboration in Microsoft Excel.

Many respondents consider education on digital platforms for PKU management very important (38.7%), extremely important (35.5%), or moderately important (12.9%). This education should specifically target not only patients but also their families (parents, partners, siblings, and grandparents), caregivers, pediatricians, social services, general practitioners, and schoolteachers/educators. Patients expressed their educational needs by suggesting cooking classes, physical activity, sports management, patient‐to‐patient exchanges on therapies, food, recipes, diet program design, and Phe calculation. These results are graphically represented in Figure 1.

As mentioned, Section 3 aimed to understand, from the patient's perspective, whether and how to include information, communication, and education in the different phases of the PCP, and through what means: face‐to‐face modalities or remote modes. Different organizational phases of the patient care pathway are conducted in a metabolic care center: the access phase; the diagnosis phase (referring to the key procedures from blood sample collection to the confirmatory diagnosis); the treatment phase (dietary phenylalanine monitoring); and the monitoring phase (periodic follow‐up with support from a multidisciplinary team—metabolic nurses, metabolic dietitian, genetic counselor, psychologist, and metabolic consultant). The follow‐up includes home blood sampling, outpatient visits, dietary assessment, and visits from multidisciplinary team members. Digital tools (e‐mail messaging, mobile apps, WhatsApp messaging) for sharing information and communicating between patients, parents, and healthcare professionals before the clinic visit could contribute to better preparing for the visit, as perceived by the respondents (74.7%).

Patients showed interest in using digital tools before their first consultation during the diagnostic process: more extensive or effective use of these tools could improve the understanding that patients, parents, or caregivers have of what healthcare professionals explain about PKU, allowing them to ask questions (80%). Additionally, the digital tools utilized by the specialized metabolic center could support the dietitian in providing information to the patient, parent, or caregiver regarding dietary treatments or condition monitoring (90.6%). During PKU daily management, digital tools could assist patients in understanding the information they receive from the metabolic team and enhancing communication outside of in‐person clinics (90.7%). For example, respondents expressed particular interest in applications for tracking PHE levels and nutritional intake (81.3%), telephone calls, and email messaging (58.7%), as well as wearable devices (49.3%).

The final part of the survey (Section 4) was co‐designed to encourage respondents to provide additional comments and suggestions and to share experiences where digital tools have assisted PKU patients, families, and caregivers with information, education, and communication in the management of PKU. Table 2 presents the detailed comments collected.

**TABLE 2:** Section 4. Final comments and suggestions from PKU patients and parents.

Information needs	“The existence of a big and reliable online platform that can be recommended by the doctors with information such as treatment, diet, low protein products, recipes that can be useful for patients, or news on any field regarding PKU, can also help a lot to obtain reliable information on an easy way for patients, families, and carers”
	“Digital tools are essential for accessing clinical trials, recording supportive data to inform HTA and to enhance clinical trial opportunities which in Ireland are abysmal. Thank you for the opportunity to comment”
Communication needs	“We live in modern world but we still lack of modern tools like blood sampling at home. Central portal to communicate with doctors, dietitians, psychologists. Would be nice to have it. Would be nice to have option to participate in clinical trials and have modern medical therapy available for all in EU”
	“More time for the patients and not always having to talk to an answering machine”
Educational needs	“Tracking app for day‐to‐day management and education into this”
	“My son thinks of a video game in which food and exercise are related and where he can earn virtual money with an ‘exemplary’ diet… Apps for calculating Phe values are very helpful, but so far, they are very manufacturer‐related”
Access	“I think that after all, the contact with persons that know how to manage PKU are the best way and the Personal contact Digital Tools should only complete this Option!”
Diagnosis	“Networking for those affected by PKU and their relatives”
Treatment	“Due to the distance from the specialistic outpatient clinics, I am only in contact once a quarter, which is sometimes really not enough, especially if your needs have changed, e.g., due to illness, sport, etc. You cannot communicate and receive support in a timely manner”
	“Real Time monitoring of PKU blood levels would be very beneficial and improve quality of life and allow for accurate treatment of PKU. Often blood levels are taken days before results are received meaning they are not true and therefore treatment is inaccurate”
	“Tracking app for day to day management and education into this”
Monitoring	“A low protein food database. Where people can list low protein foods they found in supermarkets—ideally they could input the store location to account for regional differences and make foods searchable by location”
	“At our unit we don't have access to our PHE data. We receive an SMS when it is normal, but when it is ‘abnormal’ we receive a SMS to this effect but then have to ring the hospital and try get through to a dietician. They rarely answer their phones so then leave a message for a call back, which can take days to orchestrate. Meanwhile we are anxious about the level, but without the number we are unable to action anything”
	“Own devices to determine Phe levels, similar to diabetes, e.g., useful before and during pregnancy”
	“Phe value checks at home in conjunction with an app or networking with the metabolism centre would be very helpful (home testing kits)”
	“The PHE monitor would be life changing and would help PKU patients be more self‐sufficient and save money in the long run. It would help them stick to their diet more also”

Comments indicated that digital tools are helpful for monitoring Phe levels and planning daily meals. They are also beneficial for improving communication and information sharing between the specialized metabolic center and patients, enhancing access to information on new therapies and trial opportunities, and supporting networking among PKU patients.

#### 3.2.2 Expert Healthcare Professionals' Point of View

A total of 22 respondents answered to the survey from Spain (*n* = 7, 31.9%), Germany (*n* = 4, 18.2%), UK (*n* = 4, 18.2%), Ireland (*n* = 2, 9%), The Netherlands (*n* = 2, 9%), Italy (*n* = 1, 4.6%), France (*n* = 1; 4.6%), and Turkey (*n* = 1, 4.6%). Many respondents were clinicians (*n* = 15; 68.2%) and dieticians (*n* = 7; 31.9%, including one research dietician). Almost all respondents were employed by a “specialized metabolic center.” Healthcare professionals experienced in PKU shared their insights on the key benefits for patients, families, and caregivers related to the use of digital tools during critical phases of the patient care pathway: access, diagnosis, treatment, and monitoring. They also discussed the three dimensions of information, communication, and education in managing PKU, drawing from their experience during the COVID‐19 pandemic.

Providing *information* about PKU and on *do*'s and *don't*'s was considered extremely important (63.6% of responders), primarily when directed at patients but also their families, other professionals, and schoolteachers/educators. Educational activities on the nature of PKU, synthetic protein substitutes, low‐protein foods, available therapies, and clinical trials were deemed necessary. Furthermore, healthcare professionals believed patients would benefit from being better informed in various areas, summarized in Table 3.

**TABLE 3:** Suggested topics for patient education.

Improving independence in daily life
Participating in sports
Addressing sexuality‐related issues
Educating on PKU management
Handling specific life situations such as pregnancy and maternal PKU, as well as adolescence
Inheritance of PKU
Low‐protein cooking techniques
Latest ongoing research and emerging therapies
Long‐term effects of phenylalanine control
Importance of maintaining clinical and metabolic monitoring throughout life and its impact on neurobehavior and adult mental health
Available foods and brands; the impact of aspartame in foods and medicines on PKU management
Effects of illness (such as reduced energy intake or increased energy needs) on PKU control
Role of family or support networks for individuals with PKU of any age, especially during childhood
Risks of high blood phenylalanine during pregnancy (including reasons and ways to avoid maternal PKU syndrome, importance of planned pregnancy, and preconception diet)
Lifelong transition of PKU treatment from childhood to adulthood
Importance of learning self‐care and self‐management at different stages of childhood and adolescence, along with discussions on comorbidities and patient groups

Healthcare professionals noted that patients often valued tips for specific situations, such as camping or traveling abroad, and apps for tracking phenylalanine and protein substitute intake, healthy diet, and physical exercise.

*Communication* between patients, families, caregivers, and healthcare professionals is considered extremely important (63.6% of responders) for managing PKU. Digital communication should target patients directly, along with their families, General Practitioners (GPs), other specialists, and schoolteachers or educators. Personalization of communication is considered more necessary, and individual sessions, “in person,” “by phone,” and “video calls” were suggested.

Providing *education* on a digital platform about PKU management was considered very important (40.9%) or moderately important (31.8%). This education should target patients, their families, caregivers, and their GPs. Healthcare professionals recommended educational activities, including webinars, cooking classes, training courses, and e‐learning sessions. These results are shown graphically in Figure 1.

Digital tools for sharing information and communication between patients/parents and healthcare professionals before clinic visits could improve effectiveness (77.3%). Healthcare workers consider email (77.3%) and calls (50%) to be the most useful. Telephone lines (81.8%) are the main support for patients, parents, and caregivers before their first consultation at the metabolic center. Better use of digital tools during diagnosis could enhance understanding of PKU information and opportunities for questions (81.8%). Additionally, “digital tools” employed by the specialized metabolic center could assist patients, parents, or caregivers in comprehending the dietician's information regarding dietary treatments or monitoring activities (95.5%).

**FIGURE 2:**
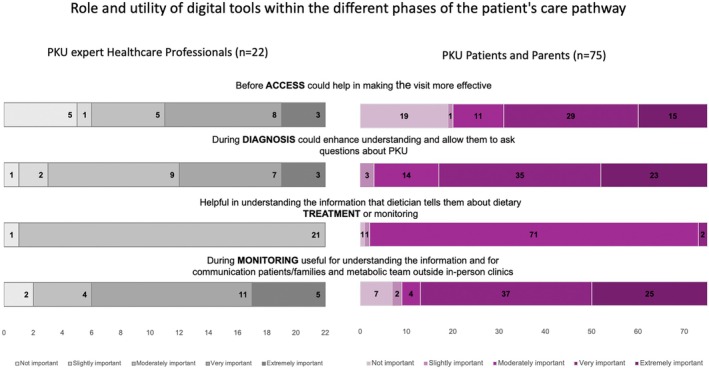
Role and utility of digital tools within the different phases of PCP. Differences and similarities between the HCP expert opinions and the patients' and parents' perspectives. *Source:* own elaboration in Microsoft Excel.

Respondents identified telephone calls (90.9%), e‐mail messaging (77.3%), apps for tracking PHE levels and nutritional intake (68.2%), and virtual calls (54.6%) as valuable resources. Figure 2 graphically illustrates the opinions of PKU HCP experts alongside the perspectives of PKU patients and parents regarding the role and utility of digital tools within the patient care pathway.

By completing the last section of the questionnaire (Section 4), healthcare professionals were invited to provide additional comments, opinions, and examples of digital tools that could aid in information, education, and communication for managing PKU. Table 4 presents collected detailed comments.

**TABLE 4:** Section 4. Final comments and suggestions from PKU expert healthcare professionals.

Information needs	“What I am really missing is information targeted at young children”
	“Regarding analysis and medical control: clear and simple information on what to do in case of possible metabolic imbalance and what to avoid; to avoid doubts about Phe”
	“During pregnancy in terms of information, and education”
Communication needs	“Direct communication is the main tool to educate patients and to help them to have the best management”
Educational needs	“Can help other personnel involved in patent's life, e.g., teachers … etc.”
	“Apps to help with cookery, recipes, shopping lists”
Access	“Patients are seen very quickly after a positive screening result. However, more info before the visit would be helpful. Otherwise, people will Google anyway”
Diagnosis	“We are surrounded by apps, smart phones and a number of means at our disposal that we do not know how to use 100%… let's turn to the possibility of creating a platform where we can go from diagnosis to our daily life in adult patients”
Treatment	“APP for the daily menu”
	“Diet apps; apps to track blood results. Apps to help with prescription ordering”
	“Easy‐to‐use apps where you can quickly calculate phe equivalents in food”
Monitoring	“Digital phe monitoring and recording would be extremely valuable and assist in the management of people with PKU greatly. It would avoid the delays between sampling and results being available”
	“As a matter of urgency, it is needed to use digital tools to help patients get their PKU medicines (amino acids, GPM protein substitutes) and their specialist low protein foods in a regular timely way, without having to do lots of explaining and phone calls to pharmacists or general/family doctors or administration staff. This is a must for patient safety.” “A way to get their blood phe results as rapidly as possible from the laboratory e.g., using whatsapp, which is secure, would be a better way almost than any other way currently available”
	“Using the electronic health records to share information with health care providers (GPs and some others…) implementing digital tools also (telemonitoring for instance) is actually working in some countries. And joining hospital pharmacists as well to monitor adherence. Finally, digital tools can be an excellent opportunity to better psychological support for PKU patients (adolescents and young adults in particular)”

## 4 Discussion

This study examines various perspectives on the potential role and utility of digital tools in managing PKU. PKU patients view digital tools as essential and valuable complements to personal care, recognizing both onsite visits and virtual care as viable modalities. Video consultations are a crucial part of telehealth and were viewed by respondents as a valuable element of the digital toolkit. Importantly, none of the suggested digital communication channels was explicitly rejected; individual video calls were favored over group video calls, and virtual and video calls can support follow‐up appointments, dietary counseling, and quick clarification of clinical issues, highlighting the viability of hybrid care approaches in PKU. One of the most exciting and innovative aspects of the research was the survey co‐design process, which allowed us to address the crucial topics deemed necessary by key stakeholders in PKU management: patients, families, caregivers, and expert healthcare professionals. The benefits of digital tools are widely acknowledged for their contributions to integrated care, proactive preventive healthcare, empowering patient communities, offsetting healthcare costs, enhancing clinical decision‐making, improving clinical trial opportunities, enhancing quality of care, and patient safety. However, drawbacks may arise if these tools are not implemented thoughtfully. Current management practices primarily focus on phenylalanine monitoring, yet PKU management must involve a holistic approach to care. It is also essential to recognize that the introduction of digital health increases the risk of over‐monitoring, such as helicopter parenting. Ensuring non‐discriminatory data protection and maintaining effective face‐to‐face communication is essential to establishing, building, and sustaining valuable, trustworthy relationships. Implementing digital tools requires early engagement with patient communities as equal partners, ensuring they are co‐designed and co‐developed to enhance utility, impact, and safety.

This study could provide insights into the perspectives, perceptions, and opinions of patients, families, and healthcare professionals through a bottom‐up approach. Utilizing digital tools to share information and communicate before the clinical visit could enhance the medical visit experience. To make the diagnosis phase more understandable and engaging, better use of digital tools could enhance patients' comprehension of what healthcare professionals communicate about PKU and offer the opportunity to ask questions and seek clarification. Digital tools could help with education as part of managing PKU.

Evidence on the clinical effectiveness and cost‐effectiveness of digital health technologies remains limited, as highlighted by recent reviews showing that, despite promising applications, real‐world adoption is still constrained, and robust comparative evaluations are lacking [23].

The phases of accessing the specialized metabolic center and the contact with specialized point of care, along with the phase of managing the main procedures for the confirmatory diagnosis, could be improved through digital tools. Parents emphasized that digital tools could help alleviate the stress they often experience during their children's diagnosis and improve understanding and communication at that time. Digital communication and information are essential for making outpatient visits more effective. Parents and adults with PKU shared comments, thoughts, and examples of how digital tools could assist with information, education, and communication in managing PKU. Healthcare providers can be essential in providing input, offering valuable information, and facilitating effective communication about the various steps and procedures in the care pathway. Digital tools could aid in conveying the dietitian's information to patient, parent, and caregiver regarding dietary treatment and monitoring of PKU.

Additionally, digital tools could enhance information and education as part of clinic visit management. These tools have the potential to enhance daily care, support tracking of Phe levels, and improve understanding of HCPs' and dieticians' perspectives. Digital tools could help gather up‐to‐date data regarding the time of the visit. This may offer several advantages, as healthcare professionals might have more valuable time during the visit, and more information could already be shared. The visit could also be more efficient and effective, as individuals are better prepared for the encounter. This is particularly important for on‐site visits with children with PKU, where interaction and informal interviews with the patient and clinician are crucial for a comprehensive assessment (Table 5).

**TABLE 5:** Main statements.

Phases of patients' care pathway	Access	Diagnosis	Treatment	Monitoring
Use of digital tools	Use of digital tools before the clinic visit	Use of digital tools before the first consultation	Use of digital tools during treatment	Use of digital tools in day‐to‐day management
Communication	Digital communication is very important to make the outpatient visit more effective and efficient	Digital communication is very important to make the first visit more effective and efficient		Digital communication tools have a high potential for improving day‐to‐day care. Digital communication tools have high potential for facilitating patients, parents, caregivers in understanding the messages of healthcare professionals and dietitians
Information	Digital information is very important to make the outpatient visit more effective and efficient	Digital information could make the diagnosis phase more clear and understandable	Information on daily implementation of nutritional therapy could be usefully supported by digital tools	Digital information on daily implementation of nutritional therapy is very important
Education		Utilization of digital tools could improve education and awareness of PKU as a chronic condition	Education on dietary management could be improved by the adoption of digital tools	PKU management education on digital platforms is important
Digital tools	Kind of digital tools: e‐mail messaging Telephone calls. Dedicated telephone lines. Mobile apps. WhatsApp messaging. Virtual calls.	Kind of digital tools: Telephone lines. e‐mail messaging. Virtual calls. WhatsApp messaging.	Kind of digital tools: e‐mail messaging. Telephone calls. Dedicated telephone lines. Mobile apps. WhatsApp messaging. Virtual calls. Digital platform for consultation in real time of personal health data and specific information.	APPs for tracking PHE‐levels are considered the most useful. Tracking PHE levels is considered very important and digital tools might help.

Differences in the perceived usefulness of specific digital tools between patients/caregivers and healthcare professionals may reflect distinct perspectives and contextual constraints. Patients and caregivers may place value on tools that facilitate daily self‐management, monitoring, and timely access to phenylalanine (Phe) results, whereas healthcare professionals may consider additional factors, such as integration into clinical workflows, data governance, privacy, and organizational requirements. Recent evidence on telemedicine in rare diseases, including inherited metabolic disorders such as PKU, indicates that digital tools can support nutritional monitoring and follow‐up, while also highlighting persistent technical and organizational challenges that limit their uniform implementation [24]. These considerations suggest that structured digital solutions for rapid Phe result delivery and monitoring represent a key unmet need and should be positioned within hybrid care models that complement face‐to‐face specialist care. Finally, digital care pathways could also benefit from linkage with established European‐level patient organizations and educational resources to support reliable information delivery and avoid fragmentation.

This work has several limitations. The sample size did not allow for statistically robust country‐level analyses, reflecting the multi‐country survey design and the focus on stakeholder perspectives rather than on single‐center clinical cohorts. The geographical distribution is influenced by the recruitment activities of national patient organizations and may therefore be better interpreted as “country snapshots” rather than a fully representative European picture. In contrast, larger samples reported in previous COVID‐19 telehealth studies in PKU [12] come from retrospective, monocentric clinical populations. Additionally, since the surveys were anonymous, it was not possible to confirm whether the same individuals participated in more than one survey, so respondent samples may vary across surveys. Lastly, educational level was not collected; therefore, the potential impact of education and digital literacy on attitudes toward and adoption of digital tools could not be evaluated and should be investigated in future research.

## 5 Conclusions

Patients with PKU, their parents, and caregivers perceive digital tools as valuable complementary resources for information, education, and communication across the patient care pathway. In particular, the use of digital tools before clinical visits is perceived as helpful for preparation, information clarification, and engagement, while face‐to‐face consultations remain essential and are not viewed as replaceable.

Our findings support the role of hybrid care models, in which digital solutions are integrated with in‐person visits to address specific needs such as education, dietary management, and monitoring support. Although this study does not assess clinical effectiveness or cost‐effectiveness, it highlights priority areas where digital tools are perceived as most useful by key stakeholders.

Despite the study's limitations, these insights could guide future research and policy discussions on using digital tools in PKU care pathways. More studies are needed to evaluate outcomes, feasibility, and long‐term effects of digital and hybrid care models in PKU management.

## Author Contributions

G.T., S.C., and V.Q. conceived the paper; S.C., L.T., and G.T. performed data analyses; S.C. wrote the first draft of the manuscript; G.T., S.C., and L.T. wrote the final draft. S.C., V.Q., B.S.‐G., T.H., A.B., S.L.D., E.P.T., A.H., A.M., E.V., J.O., M.S., F.V.S., E.L., T.B., A.M., S.v.D., L.T., and G.T. were involved in study design and discussion, attended all stakeholders meetings, and interpreted the results. B.S.‐G., T.H., and A.B. distributed the patient‐parent caregiver survey. S.C., V.Q., T.H., A.B., S.L.D., E.P.T., A.H., A.M., E.V., J.O., M.S., F.V.S., E.L., T.B., A.M., S.v.D., L.T., and G.T. repeatedly edited the manuscript and approved the submitted version. G.T. is the corresponding author.

## Funding

Funding from the European Brain Council is acknowledged.

## Ethics Statement

The adopted approach was for patients to fill out the anonymous surveys (distributed by Patient Associations among their members) with an explicit consent statement; the IRB was not requested. PKU patient representatives were involved in the work as study group members.

## Conflicts of Interest

The authors declare no conflicts of interest.

## Supporting information


**Supplementary File 1.** Survey questionnaire for patients and caregivers.

**Supplementary File 2.** Survey questionnaire for healthcare professionals.

## Data Availability

The data supporting this study's findings are included in this published article and are also available in the Supporting Information accompanying this article.
