# Supplementary material for: The Role of Digital Tools and Their Implementation Within Patient Care Pathways for Rare Brain Disorders: The Case of Phenylketonuria
Source: Eur J Neurol. 2026 Apr 9;33(4):e70575. doi: 10.1111/ene.70575 (PMC13062938; doi:10.1111/ene.70575)
Supplement: Supplementary file 1 — Survey questionnaire for patients and caregivers. [file ENE-33-e70575-s001.pdf]

“This questionnaire is the intellectual property of the authors and is provided for research transparency. Any reproduction, distribution, adaptation, or use of this material requires prior written permission from corresponding author. If permission is granted, the original source must be properly cited.”

WHERE AND HOW CAN DIGITAL TOOLS SUPPORT PKU DIAGNOSIS, TREATMENT AND FOLLOW-UP?

Help us to learn more!

We truly appreciate your time and effort, and we are excited to hear your honest feedback!

PRIVACY STATEMENT.

The participation in this survey is voluntary.

The survey is conducted by a research group composed of the European Brain Council together with its academic partner (Sant’Anna School of Advanced Studies - Pisa, Italy) and the representatives of PKU National Patients Associations. The main aim of the survey is to understand the needs of patients with Phenylketonuria and to define the best channels to engage and communicate with them.

The link to the survey is exclusively sent by the National PKU Associations.

The survey is intended to be anonymous, and all efforts have been made to ensure anonymity. The responses to the survey were aggregated in a manner that neither allowed for the identification of individual respondents nor for the attribution of individual responses to a respondent.

The research team will not be able to identify the respondent. Responses in the open-space fields should therefore be formulated in such a way that no information permitting identification of the respondent, or another person is introduced. The anonymized and aggregated data will be kept for a maximum period of one year after the closure of the survey.

Fields marked with * are mandatory.

☐ I DECLARE that I have read and fully understood the “Privacy Statement”. I also declare that I have read and understood the purposes and methods of processing personal data and that I have had sufficient time to decide, freely and voluntarily, whether or not to give consent

A. PKU and YOU

* Who are you?
  - ○ I’m a person with PKU (fenilcetonuria)
  - ○ I’m a parent (or a guardian) of a person with PKU
  - ○ I’m a spouse / partner of a person with PKU
  - ○ I’m a sibling of a person with PKU
  - ○ I’m a grandparent of a person with PKU
  - ○ I’m a carer of a person with PKU

* Your age
  - ○ 18 - 20
  - ○ 21 - 35
  - ○ 36-45
  - ○ 46 - 60
  - ○ More than 60

* Your gender
  - ○ Male
  - ○ Female
  - ○ I do not wish to say

* Please, select the situation that best represents the person with PKU.

(If you are a parent, a spouse/partner, a sibling, a grandparent, or a carer, in this question, please, answer for the person with PKU you are caring for)

PKU DIAGNOSIS: when your healthcare professional tells you about the confirmatory diagnostic of PKU.
  - ○ I had a PKU diagnosis when I was a baby (newborn)
  - ○ I had a PKU diagnosis when I was a child (between 3 months and 7 years old)
  - ○ I had a PKU diagnosis when I was 7 years old or older

* Does your PKU require treatment (e.g. classical PKU) or is it a mild form like Hyperphenylalaninemia that does not require treatment?

(If you are a parent, a spouse/partner, a sibling, a grandparent, or a carer, in this question, please, answer for the person with PKU you are caring for)

PKU TREATMENT : the dietary phenylalanine restriction/monitoring.
  - ○ Treatment required
  - ○ No treatment required

* Are you currently on dietary or any other treatment?

(If you are a parent, a spouse/partner, a sibling, a grandparent, or a carer, in this question, please, answer for the person with PKU you are caring for)

- Yes
- No

* Are you currently followed up by a specialised metabolic centre?

(If you are a parent, a spouse/partner, a sibling, a grandparent, or a carer, in this question, please, answer for the person with PKU you are caring for)

TO BE IN FOLLOW-UP : collecting home blood sampling, and when periodically you go to the hospital for periodic outpatient visits, dietary assessment, multidisciplinary visits (for example: psychologist)

- Yes
- No

* Country where you live

- Germany
- Republic of Ireland
- Spain
- Other

B. INFORMATION, COMMUNICATION and EDUCATIONAL NEEDS

* **1. Do you think that providing information about PKU and what about to do/don't do is important?**

INFORMATION : giving advice about PKU

- Yes
- No
- I don't know

* In your opinion, activities that provide information on PKU should be targeted at:

(multiple choice is available)

INFORMATION ACTIVITIES: : giving advice about how to book an appointment for clinic visits, about where the specialised metabolic centre is located, how to arrive to the specialised medical centre, about existing PKU patients' associations, etc.

- [ ] Persons with PKU
- [ ] Parent (or guardian)
- [ ] Spouse / partner
- [ ] Sibling
- [ ] Grandparent
- [ ] Carer

* Which kind of information you think could be useful?

(multiple choice is available)

CLINICAL TRIAL : clinical research study also involving patients and clinicians, for evaluation of the efficacy of new drugs or new medical devices.

- [ ] Information on the nature of PKU
- [ ] Medical Foods available to you
- [ ] Therapies available to you
- [ ] Clinical trial opportunities
- [ ] Specialised metabolic centre near to you
- [ ] Synthetic Protein Substitutes available to you
- [ ] Other

Other, please tell us

* Please specify other areas that you would like to be more informed on from your specialised metabolic centre.

SPECIALISED METABOLIC CENTRE : a metabolic care unit or metabolic care center that is specialised in the care of PKU.

*** 2. Do you think that communication between patient/families/carers and healthcare professionals is important for the management of PKU?**

COMMUNICATION : sharing of information with someone

HEALTHCARE PROFESSIONAL : doctor, nurse, dietician, physiotherapist, etc.

- ○ Yes
- ○ No
- ○ I don't know

If yes, please rate how important it is for PKU.

- ○ Not important at all
- ○ Slightly important
- ○ Moderately important
- ○ Very important
- ○ Extremely important

* In your opinion, the communication activities should be addressed to:

(multiple choice is available)

- [ ] Persons with PKU
- [ ] Parent (or a guardian)
- [ ] Spouse / partner
- [ ] Sibling
- [ ] Grandparents
- [ ] Carer
- [ ] Other

Other, please specify

* Which kind of communication channels you think could be useful?

(multiple choice is available)

- [ ] In person
- [ ] By telephone calls
- [ ] By text messages
- [ ] Via videocalls group sessions
- [ ] Via videocall individual sessions
- [ ] Other

Other, please specify

* How do you feel the communication between you and your specialised metabolic centre can improve?

*** 3. Do you think that providing education on a digital platform about the management of PKU is important?**

EDUCATION: improve the knowledge about PKU.

- ( ) Yes
- ( ) No
- ( ) I don't know

* If yes, please rate how important it is for PKU.

- Not important at all
- Slightly important
- Moderately important
- Very important
- Extremely important

* In your opinion, the educational activities on a digital platform should be targeted at:

(multiple choice is available)

EDUCATIONAL ACTIVITIES : such as webinars, training courses, e-learning courses, leaflets, newsletter

- Persons with PKU
- Parent (or a guardian)
- Spouse / partner
- Sibling
- Grandparent
- Carer
- Other

Other - please, specify

* Which kind of educational activities you think could be organised with the use of a digital platform?

(multiple choice is available)

- Webinars
- Training courses
- E-learning courses
- Educational programs
- Other

Other - please, specify

* What other educational activities do you think could be included with the use of a digital platform?

* **4. Which digital tools do you currently use? (Apps, internet information, virtual communication with doctors/dieticians, social networks, etc.)**

C. ACCESS, DIAGNOSIS, TREATMENT and MONITORING of YOUR PKU.

(If you are a parent, a spouse/partner, a sibling, a grandparent, or a carer, in the following questions you will be asked to answer for the person with PKU you are caring for)

1. ACCESS

The phase of the contact with the specialised metabolic centre (a clinic or a centre that is experienced with PKU care).

* Do you have access to a specialised metabolic centre?

- Yes
- No

* During the COVID 19 pandemic (during 2020 and 2021), did you have physical access to the specialised metabolic centre?

- Yes
- No

* During the pandemic, did you experience

(multiple choice is available)

- An interruption of planned visits
- An interruption of regular outpatient's clinic services - such as blood Phe monitoring and assessment of nutritional treatments according to blood Phe levels
- Supplement supply issues
- Other

* How do you usually communicate with your specialised metabolic centre (or your case manager)?

(multiple choice is available)

- By telephone
- By e-mail
- In person
- Virtual call

* During the COVID 19 pandemic (during 2020 and 2021), did you communicate differently with your specialised metabolic centre?

- Yes
- No

* How did you communicate with your specialised metabolic centre during the Covid 19 pandemic?

(multiple choice is available)

- [ ] Long telephone calls
- [ ] Video - consultations
- [ ] Other

Other - Please, tell us

* And how often did you communicate with your specialised metabolic centre during the Covid 19 pandemic?

- Once a year
- Twice a year
- Every 3 months
- Each month
- More than once a month
- Weekly

* Do you think that the use of digital tools for sharing information and for communicating between you and the healthcare professionals **before** your clinic visit could help in making the visit more effective?

- Yes
- No
- I don't know

* In your opinion, which kind of digital tools could be used **before** your clinic visit to the specialised metabolic centre?

(multiple choice is available)

- [ ] Dedicated telephone lines
- [ ] Telephone calls
- [ ] E-mail messaging
- [ ] Virtual calls
- [ ] WhatsApp messaging
- [ ] Mobile Apps

* What prohibits your engagement with digital tools. Please, tell us.

2. DIAGNOSIS

DIAGNOSIS of PKU refers to the main procedures from blood sample until the confirmatory diagnosis that you experienced at the clinic you were initially referred to for specialised care (specialised metabolic centre or any other healthcare facility).

At the point of diagnosis

* What kind of digital tools could be used by your specialised metabolic centre to support you **before** you or your family member’s first consultation?

(multiple choice is available)

- ☐ Telephone lines
- ☐ E-mail messaging
- ☐ WhatsApp messaging
- ☐ Virtual calls

* What kind of digital tools could have helped you in making the diagnosis phase more understandable and effective? Were those tools available or not?

* Do you think that a more extensive/better use of digital tools could enhance your understanding of what healthcare professionals tell you about PKU and afford you the opportunity to ask questions about PKU?

- ○ Yes
- ○ No
- ○ I don't know

* Please rate how important it is for PKU.

- ○ Not important at all
- ○ Slightly important
- ○ Moderately important
- ○ Very important
- ○ Extremely important

3. TREATMENT

TREATMENT for PKU (dietary phenylalanine monitoring).

* **In terms of treatment**, do you think that digital tools could be useful for understanding the information that your dietician tells you about the dietary treatment or monitoring?

- Yes
- No
- I don't know

* In your opinion, which digital tools could be adopted by your specialised metabolic centre that could help the patient / family with the day to day management of PKU?

(multiple choice is available)

- Telephone calls
- E-mail messaging
- Virtual calls
- WhatsApp messaging
- Mobile apps
- Other

Other - Please, tell us

---

4. MONITORING

MONITORING of PKU: : the periodic follow-up with the support of a multidisciplinary team (metabolic nurses, metabolic dietitian, genetic counsellor, psychologist and metabolic consultant) for both children and adults and a specialised metabolic laboratory. The follow-up includes home blood sampling, outpatient visits, dietary assessment, a multidisciplinary team member visit (for example: psychologist).

* Day to day PKU Management: Do you think that digital tools could be useful for understanding the information you receive from your metabolic team and for communication between you and your Metabolic team outside of your in-person clinics?

- Yes
- No
- I don't know

* In your opinion, which kind of digital tools could be used **during** the follow-up phase?

(multiple choice is available)

- Telephone calls
- E-mail messaging
-

WhatsApp messaging
- [ ] Virtual calls
- [ ] Wearable devices
- [ ] An application to track PHE levels and nutritional intake

---

* **5. Would you like to add other comments and thoughts, and/or provide examples where you feel that digital tools could help you in terms of information, education, communication as part of the management of PKU?**

Contact

sara.cannizzoatgmail.com
