# Supplementary material for: The Role of Digital Tools and Their Implementation Within Patient Care Pathways for Rare Brain Disorders: The Case of Phenylketonuria
Source: Eur J Neurol. 2026 Apr 9;33(4):e70575. doi: 10.1111/ene.70575 (PMC13062938; doi:10.1111/ene.70575)
Supplement: Supplementary file 2 — Survey questionnaire for healthcare professionals. [file ENE-33-e70575-s002.pdf]

"This questionnaire is the intellectual property of the authors and is provided for research transparency. Any reproduction, distribution, adaptation, or use of this material requires prior written permission from corresponding author. If permission is granted, the original source must be properly cited."

WHERE AND HOW CAN DIGITAL TOOLS SUPPORT PKU DIAGNOSIS, TREATMENT AND FOLLOW-UP?

Help us to learn more!

We truly appreciate your time and effort, and we are excited to hear your honest feedback!

PRIVACY STATEMENT.

The participation in this survey is voluntary.

The survey is conducted by a research group composed by the European Brain Council together with its academic partner (Sant'Anna School of Advanced Studies - Pisa, Italy) and the representatives of PKU National Patients Associations. The main aim of the survey is to collect the opinions of the expert clinicians and other expert healthcare professionals about their and PKU patients' unmet needs on information, communication, and education and to define the best channels to engage and communicate with them.

Fields marked with * are mandatory.

☐ I DECLARE that I have read and fully understood the “Privacy Statement”. I also declare that I have read and understood the purposes and methods of processing personal data and that I have had sufficient time to decide, freely and voluntarily, whether or not to give consent

A. PKU and YOU

* Who are you?
  - ○ I’m a clinician
  - ○ I’m a dietician
  - ○ I’m a nurse
  - ○ Other

* Your age
  - ○ 18 - 20
  - ○ 21 - 35
  - ○ 36-45
  - ○ 46 - 60
  - ○ More than 60

* Your gender
  - ○ Male
  - ○ Female
  - ○ I do not wish to say

* Country where you live
  - ○ Germany
  - ○ Republic of Ireland
  - ○ Spain
  - ○ Other

* Are you currently employed by a specialised metabolic centre?

SPECIALISED METABOLIC CENTRE : a metabolic care unit or metabolic care center that is specialised in the care of PKU.

  - ○ Yes
  - ○ No

B. INFORMATION, COMMUNICATION and EDUCATIONAL PATIENT' NEEDS

* **1. Do you think that providing information about PKU and what about to do/don’t do is important?**

INFORMATION : giving advice about PKU

- Yes
- No
- I don't know

* In your opinion, activities that provide information on PKU should be targeted at:

(multiple choice is available)

INFORMATION ACTIVITIES: giving advice about how to book an appointment for clinic visits, about where the specialised metabolic centre is located, how to arrive to the specialised medical centre, about existing PKU patients' associations, etc.

- [ ] Persons with PKU
- [ ] Parent (or guardian)
- [ ] Spouse / partner
- [ ] Sibling
- [ ] General Pratictioner
- [ ] Caregiver
- [ ] Other

* Which kind of information do you think could be useful?

(multiple choice is available)

CLINICAL TRIAL : clinical research study also involving patients and clinicians, for evaluation of the efficacy of new drugs or new medical devices.

- [ ] Information on the nature of PKU
- [ ] Medical Foods available to the patient
- [ ] Therapies available to the patient
- [ ] Clinical trial opportunities
- [ ] Specialised metabolic centre near to the patient
- [ ] Synthetic Protein Substitutes available to the patient
- [ ] Other

* Please specify other areas that you think the patient might like to be more informed on.

---

* **2. Do you think that communication between patient/families/carers and healthcare professionals is important for the management of PKU?**

COMMUNICATION : sharing of information with someone

HEALTHCARE PROFESSIONAL : doctor, nurse, dietician, physiotherapist, etc.

- Yes

- No
- I don't know

* In your opinion, digital communication should be target at:

(multiple choice is available)

- Persons with PKU
- Parent (or a guardian)
- Spouse / partner
- Sibling
- Carergiver
- General Pratictioner
- Other

* Which kind of communication channels you think could be useful?

(multiple choice is available)

- In person
- By telephone calls
- By text messages
- Via videocalls group sessions
- Via videocall individual sessions
- Other

* How do you feel the communication between the patients/parents/carers and the specialised metabolic centre can improve?

*** 3. Do you think that providing education on a digital platform about the management of PKU is important?**

EDUCATION: improve the knowledge about PKU.

- Yes
- No
- I don't know

* If yes, please rate how important it is.

- Not important at all
- Slightly important
- Moderately important
- Very important
- Extremely important

* In your opinion, the educational activities on a digital platform should be targeted at:

(multiple choice is available)

EDUCATIONAL ACTIVITIES : such as webinars, training courses, e-learning courses, leaflets, newsletter

- [ ] Persons with PKU
- [ ] Parent (or a guardian)
- [ ] Spouse / partner
- [ ] Sibling
- [ ] Caregiver
- [ ] General Pratictioner
- [ ] Other

* Which kind of educational activities you think could be organised with the use of a digital platform?

(multiple choice is available)

- [ ] Webinars
- [ ] Training courses
- [ ] E-learning courses
- [ ] Educational programs
- [ ] Other

* What other educational activities do you think could be included with the use of a digital platform?

---

* **4. Which digital tools do you currently use? (Apps, virtual communication with colleagues, social networks, etc.)**

C. ACCESS, DIAGNOSIS, TREATMENT and MONITORING of PKU.

1. ACCESS
The phase of the patient contact with the specialised metabolic centre (a clinic or a centre that is experienced with PKU care).

* During the COVID 19 pandemic (during 2020 and 2021), did patients have physical access to the specialised metabolic centre?

- ( ) Yes
- ( )

No

* During the pandemic, did PKU patients experience

(multiple choice is available)

- [ ] An interruption of planned visits
- [ ] An interruption of regular outpatient's clinic services - such as blood Phe monitoring and assessment of nutritional treatments according to blood Phe levels
- [ ] Supplement supply issues
- [ ] Other

* How does the patient usually communicate with the specialised metabolic centre (or the case manager)?

(multiple choice is available)

- [ ] By telephone
- [ ] By e-mail
- [ ] In person
- [ ] Virtual call
- [ ] Other

* During the COVID 19 pandemic (during 2020 and 2021), did the patient communicate differently with the specialised metabolic centre?

- ( ) Yes
- ( ) No

* How did the patient communicate with the specialised metabolic centre during the Covid 19 pandemic?

(multiple choice is available)

- [ ] Long telephone calls
- [ ] Video - consultations
- [ ] Other

* And how often did the patient communicate with the specialised metabolic centre during the Covid 19 pandemic?

- ( ) Once a year
- ( ) Twice a year
- ( ) Every 3 months
- ( ) Each month
- ( ) More than once a month
- ( ) Weekly

* Do you think that the use of digital tools for sharing information and for communicating between the patient and the healthcare professionals **before** the clinic visit could help in making the visit more effective?

- ( ) Yes
- ( ) No
- ( )

I don't know

* In your opinion, which kind of digital tools could be used **before** the clinic visit to the specialised metabolic centre?

(multiple choice is available)

- [ ] Dedicated telephone lines
- [ ] Telephone calls
- [ ] E-mail messaging
- [ ] Virtual calls
- [ ] WhatsApp messaging
- [ ] Mobile Apps
- [ ] Other

* In your opinion, what prohibits the patient/parent/carers engagement with digital tools.

---

2. DIAGNOSIS

DIAGNOSIS of PKU refers to the main procedures from blood sample until the confirmatory diagnosis that the patient experiences at the clinic he/she was initially referred to for specialised care (specialised metabolic centre or any other healthcare facility).

At the point of diagnosis

* What kind of digital tools could be used by the specialised metabolic centre to support the patient/parents /caregivers **before** the first consultation?

(multiple choice is available)

- [ ] Telephone lines
- [ ] E-mail messaging
- [ ] WhatsApp messaging
- [ ] Virtual calls
- [ ] Other

* What kind of digital tools could help the patients in making the diagnosis phase more understandable and effective? Are those tools available or not?

* Do you think that a more extensive/better use of digital tools could enhance patient/parent/caregiver understanding of what healthcare professionals tell him/her about PKU and afford his/her the opportunity to ask questions about PKU?

- Yes
- No
- I don't know

* Please rate how important it is for PKU.

- Not important at all
- Slightly important
- Moderately important
- Very important
- Extremely important

---

3. TREATMENT

TREATMENT for PKU (dietary phenylalanine monitoring).

* **In terms of treatment**, do you think that digital tools could be useful for understanding the information that the dietician provides to the patient/parent/caregiver about the dietary treatment or monitoring?

- Yes
- No
- I don't know

* In your opinion, which digital tools could be adopted by the specialised metabolic centre that could help the patient/family with the day-to-day management of PKU?

(multiple choice is available)

- Telephone calls
- E-mail messaging
- Virtual calls
- WhatsApp messaging
- Mobile apps
- Other

---

4. MONITORING

MONITORING of PKU: the periodic follow-up with the support of a multidisciplinary team (metabolic nurses, metabolic dietician, genetic counsellor, psychologist and metabolic consultant)

for both children and adults and a specialised metabolic laboratory. The follow-up includes home blood sampling, outpatient visits, dietary assessment, a multidisciplinary team member visit (for example: psychologist).

* Day to day PKU Management: Do you think that digital tools could be useful for understanding the information patient/parents/caregivers receive from the metabolic team and for communication between patient/parents /caregivers and the metabolic team outside of the in-person clinics?

- ○ Yes
- ○ No
- ○ I don't know

* In your opinion, which kind of digital tools could be used **during** the follow-up phase?

(multiple choice is available)

- ☐ Telephone calls
- ☐ E-mail messaging
- ☐ WhatsApp messaging
- ☐ Virtual calls
- ☐ Wearable devices
- ☐ An application to track PHE levels and nutritional intake

* **5. Would you like to add other comments and thoughts, and/or provide examples where you feel that digital tools could help in terms of information, education, communication as part of the management of PKU?**

* **6. In your country, is there a national plan for digitalisation of healthcare processes, like: telemedicine platforms, Apps for remote monitoring of health parameters, digitalisation of healthcare pathways, etc.**
